# Genomes of Abundant and Widespread Viruses from the Deep Ocean

**DOI:** 10.1128/mBio.00805-16

**Published:** 2016-07-26

**Authors:** Carolina Megumi Mizuno, Rohit Ghai, Aurélien Saghaï, Purificación López-García, Francisco Rodriguez-Valera

**Affiliations:** a Evolutionary Genomics Group, Universidad Miguel Hernandez, Alicante, Spain; b Unit of Molecular Biology of the Gene in Extremophiles, Department of Microbiology, Institut Pasteur, Paris, France; c Department of Aquatic Microbial Ecology, Biology Center of the Academy of Sciences of the Czech Republic, Institute of Hydrobiology, České Budějovice, Czech Republic; d Unité d’Ecologie, Systématique et Evolution, CNRS UMR 8079, Université Paris-Sud, Orsay, France

## Abstract

The deep sea is a massive, largely oligotrophic ecosystem, stretched over nearly 65% of the planet’s surface. Deep-sea planktonic communities are almost completely dependent upon organic carbon sinking from the productive surface, forming a vital component of global biogeochemical cycles. However, despite their importance, viruses from the deep ocean remain largely unknown. Here, we describe the first complete genomes of deep-sea viruses assembled from metagenomic fosmid libraries. “*Candidatus* Pelagibacter” (SAR11) phage HTVC010P and *Puniceispirillum* phage HMO-2011 are considered the most abundant cultured marine viruses known to date. Remarkably, some of the viruses described here recruited as many reads from deep waters as these viruses do in the photic zone, and, considering the gigantic scale of the bathypelagic habitat, these genomes provide information about what could be some of the most abundant viruses in the world at large. Their role in the viral shunt in the global ocean could be very significant. Despite the challenges encountered in inferring the identity of their hosts, we identified one virus predicted to infect members of the globally distributed SAR11 cluster. We also identified a number of putative proviruses from diverse taxa, including deltaproteobacteria, bacteroidetes, SAR11, and gammaproteobacteria. Moreover, our findings also indicate that lysogeny is the preferred mode of existence for deep-sea viruses inhabiting an energy-limited environment, in sharp contrast to the predominantly lytic lifestyle of their photic-zone counterparts. Some of the viruses show a widespread distribution, supporting the tenet “everything is everywhere” for the deep-ocean virome.

## IMPORTANCE

The deep sea is among the largest known habitats and a critical cog in biogeochemical cycling but remains underexplored in its microbiology. Even more than is the case for its prokaryotic community, our knowledge of its viral component has remained limited by the paucity of information provided by studies dependent upon short sequence fragments. In this work, we attempt to fill this existing gap by using a combination of classical fosmid libraries with next-generation sequencing and assembly to recover long viral genomic fragments. We have sequenced ca. 6,000 fosmids from two metagenomics libraries made from prokaryotic biomass from the deep Mediterranean Sea and recovered twenty-eight complete viral genomes, all of them novel and quite distinct from all previously described viral genomes. They are preferentially found in deeper waters and are widely distributed all over the oceans. To our knowledge, this is the first report on complete and cosmopolitan viral genomes from the bathypelagic habitat.

## INTRODUCTION

The role of marine viruses in the carbon cycle and in maintaining diversity in bacterial, archaeal, and eukaryotic populations in the ocean is critical (1–3). Metagenomics has played an important role in unveiling the enormous range of phylogenetic and metabolic diversity of the ocean microbiome (4–7). However, the difficulties of studying viruses are daunting. Obtaining axenic cultures of marine prokaryotes is the most significant obstacle that must be overcome in order to isolate individual viruses, as the vast majority of abundant microbes remain uncultured. Metagenomic fosmids have recently appeared as a promising alternative to retrieve complete genomes of uncultured viruses, as they can contain significant amounts of viral DNA derived from cells undergoing the lytic cycle (4, 5, 8, 9). In particular, the concatemer replication phase of the caudovirales, frequently infecting marine plankton, represents a natural way of amplifying these viral genomes within their natural hosts. Given that fosmid insertions can be up to 40 kb in length and that the average genome length of podoviruses and siphoviruses (both caudovirales) is ca. 50 kb, retrieval of full-genome sequences for these groups is feasible. Applying this strategy, Mizuno et al. were able to describe more than 1,000 viral genomic fragments that were more than 10 kb in length from a fosmid library from the Mediterranean deep chlorophyll maximum (MedDCM), of which 208 represented complete viral genomes (10). In the case of the even more unexplored deep sea (11), only a few metagenomic snapshots into the metabolic potential of some deep-sea planktonic microbes such as archaea (12–14) and *Acidobacteria* (15) are available. If deep-sea prokaryotes are poorly understood, their viruses remain practically unknown. Most viral genomes from the deep-sea habitat that have been described are associated with hydrothermal vents, e.g., Panulirus argus virus 1 (PaV1) infecting the euryarchaeon *Pyrococcus abyssi* (16) and the recently described viral genomes that encode sulfur oxidation genes (17). However, hydrothermal vents are highly specialized habitats and are not representative of the deep ocean at large. There are also a few examples of non-vent-associated phages, e.g., a bacteriophage infecting *Aurantimonas*, a psychrotolerant *Alphaproteobacteria* species isolated from bathypelagic waters (18), and a filamentous phage infecting *Shewanella piezotolerans*, isolated from marine sediment (19). However, the vast majority of viruses infecting abundant bathypelagic microbiota remain unexplored and some insights have only recently been made possible using metaviromes, i.e., direct sequencing of nucleic acids extracted from size-fractionated viral particles (virions) (20–22). The Pacific Ocean virome (POV) data set, comprising 12 samples from the deep Pacific (1,000 to 4,300 m in depth), offered the first glimpses of viruses from the bathypelagic realm (20). In comparison to viromes from the photic zone, these deep viromes revealed a distinct set of auxiliary metabolic genes, indicating the presence of niche-defining functions across depths (23). In addition, deep-sea viromes from the Atlantic Ocean and the Mediterranean Sea showed very similar diversity patterns in two geographically distant locations (21). However, despite the information derived from these direct metavirome approaches, large assemblies providing reference genomes from deep-sea viruses are still missing.

In this work, we have applied a fosmid-based viral genome recovery approach to the deep-ocean plankton. We have sequenced metagenomic fosmids from two deep (1,000-m and 3,000-m) Mediterranean Sea metagenomic libraries (6, 14) to obtain the first glimpses of the genomic repertoire of viruses infecting deep-ocean plankton. We describe the first complete viral genomes from deep-sea waters, some of which belong to completely novel viral lineages that appear to be widespread and abundant worldwide.

## RESULTS

### uvDeep: viral genomic fragments from bathypelagic waters.

In this work, we have sequenced 6,432 fosmids from two metagenomic libraries constructed from deep-sea Mediterranean plankton (3,168 clones from AD1000 [Adriatic Sea; 1,000-m depth] and 3,264 clones from KM3 [Ionian Sea; 3,000-m depth]). Their assembly yielded 8,000 contigs larger than 10 kb, ca. 3,000 of which appeared to be complete, as both contained fosmid-end sequences, suggesting a high (46%) rate of recovery of complete fosmids and supporting a nonchimeric assembly. In our previous work at the MedDCM, more than 1,000 contigs of viral origin (referred to here as uvMED), 208 of which represented complete viral genomes, were retrieved (24). These figures are not surprising, considering that, in the deep chlorophyll maximum of tropical and temperate seas, around 10% to 15% of clones in metagenomic libraries built from prokaryotic biomass derive from viral DNA (4, 5).

Of the ca. 6,000 bathypelagic fosmids, sequence annotations suggested that 99 contigs were of obvious viral origin (and are referred to here as uvDeep [see Table S1 in the supplemental material]). Of these, 75 (representing only ca. 1.1%, including complete genomes and incomplete fragments) were predicted to originate from head-tail viruses as determined by the presence of characteristic proteins, e.g., terminases, portal proteins, or tail proteins. Another 10 contigs related to head-tailed viruses were identified as proviruses by the presence of flanking or adjoining tracts of genes from microbial genomes (see Table S2). However, nearly all of these were found to be singletons, i.e., to have no close relatives among even the fosmids sequenced from the same location. Only six genomes showed significant similarity to uvMED genomes (see Fig. S1 and Fig. S2).

**FIG 1:**
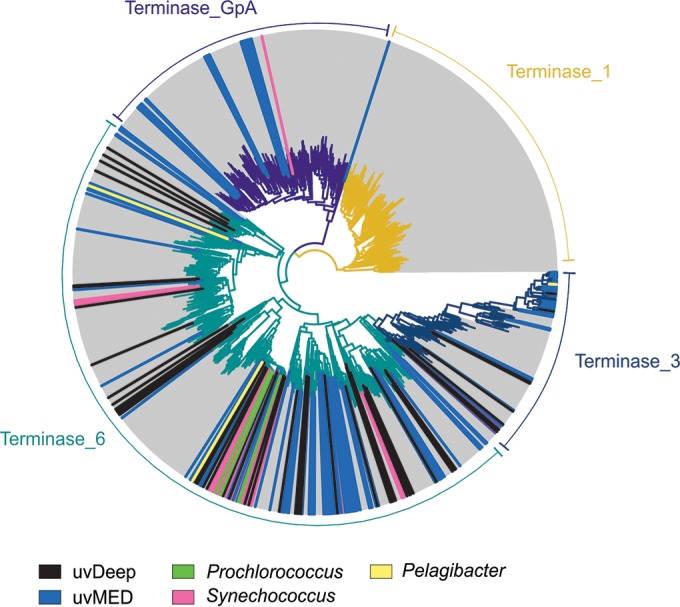
Terminase phylogeny. A maximum likelihood phylogenetic tree of the four major types of phage terminase large-subunit domains is shown. The terminase sequences from this study (uvDeep) are indicated by black lines, and additional sequences (uvMED) from a previous metagenomics study are in blue. Sequences from cultured cyanophages (*Prochlorococcus* and *Synechococcus*) and pelagiphages are also marked (see color legend at the bottom).

To better assess the diversity of those genomic fragments predicted to represent head-tailed viruses, we constructed a phylogenetic tree using the large-subunit terminase domain (Fig. 1). It is apparent that the deep terminases correspond to a number of novel lineages. However, only terminase_6 and the related terminase_3 type domains were found in the uvDeep data set, suggesting a somewhat limited diversity of phage terminase types in samples from bathypelagic waters in comparison to those from the DCM.

Of the remaining non-caudovirales contigs, 13 were related to viruses infecting eukaryotes. Of these, 12 had a majority of hits corresponding to the giant virus of *Phaeocystis globosa* from the *Phycodnaviridae* family (25). Viruses from this family are characterized by their replication within the host nucleus (25–27) and usually have large genomes of between 200 and 500 kb. The other contig originating from a eukaryotic virus appeared to belong to the family *Iridoviridae*. These are also giant viruses with linear genomes of 140 to 303 kb and are known to infect metazoans (28). Overall, the contigs of eukaryotic viruses recovered here were not longer than 40 kb (with an average length of 20 kb) and, in sharp contrast to head-tail viruses, did not recruit any reads from any virome or metagenome (see below and Materials and Methods). Moreover, despite the retrieval of long contigs, it was not possible to connect the genomic fragments into larger scaffolds, limiting further analyses of these sequences.

### Complete viral genomes from the deep ocean.

Of the 75 viral contigs belonging to the caudovirales, 28 were identified as complete viral genomes by the presence of repeated sequences (>50 bp) at their ends resembling a circle-like structure (10) that suggests that all genes within these genomes have been captured. The lengths of these complete viral genomes ranged from 29.8 to 41.2 kb, with GC content ranging from 30% to 61% (see Table S1 in the supplemental material). These are the first complete viral genomes to have been described by culture-independent approaches using samples from bathypelagic waters and among the very few presently described regardless of the method. Figure S1 shows an all-versus-all protein comparison of all 28 of the complete novel uvDeep genomes. As mentioned above, most of these genomes represent singletons with no sequence similarity. Only nine could be assigned to groups (group 1, group 2, and group 3, with five, two, and two representatives, respectively).

**FIG 2:**
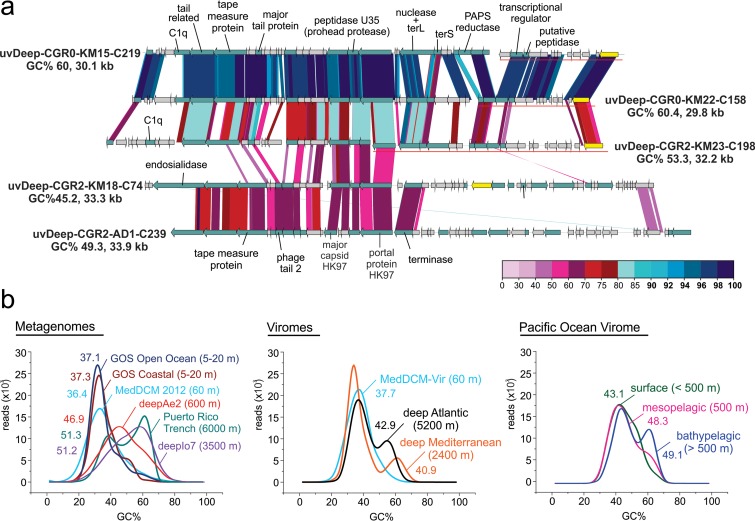
(a) Overview of genomic synteny and similarities between complete genomes from group 1. A color scale for percent identity (protein) is shown at the bottom right. The name, percent GC content (GC%), and length for each genome are also indicated. Some gene clusters are shown displaced and underlined in the graphic, indicating that they have been moved to improve comparisons across all genomes. (b) GC plot of metagenomes and viromes from different depths; the mean GC% is also shown for each of the datasets.

An alignment of group 1 genomes is shown in Fig. 2a. They harbor genes encoding a tape measure protein, suggesting they are either sipho- or myoviruses. Despite the relatively low (~50%) protein similarity, the results from the single AD1000 group 1 viral genome recovered show that most of the structural proteins are conserved and syntenic compared to those from KM3 while the rest of the genome appears to be much more divergent. All four viral genomes from KM3 encode a viral integrase, suggesting a lysogenic lifestyle. Group 1 genomes appear to have relatively high (45% to 60%) GC content. The GC content of metagenomes tends to increase with depth in the marine habitat. We observed this trend for both cells (metagenomes) and viruses (viromes) (Fig. 2b). In fact, samples from deeper waters exhibited a bimodal GC content distribution in metagenomes and viromes, with low and high peaks of GC content, while those from the photic zone appear to have only a peak of low GC. The results of comparisons of viromes from the POV divided by depth (surface, mesopelagic, and bathypelagic; see Materials and Methods for details) show a similar pattern.

In order to evaluate the novelty of uvDeep viruses, we also performed all-versus-all whole-genome comparisons of the complete viral genomes to available viral reference genomes, including those from the uvMED data set (24) and a number of other marine references (see Materials and Methods). The results indicate that while most of the uvDeep viruses are completely novel, suggesting the presence of a unique viral community in bathypelagic waters, a few appear to be distantly related to previously described viruses (e.g., pelagiphage HTVC010P and some uvMED viruses) (see Fig. S2 and S3 in the supplemental material). We have recovered 28 uvDeep complete caudoviral genomes (~0.5% from the 6,000 fosmids sequenced), a 6-fold decrease from the 208 uvMED complete caudoviral genomes (~3% of 7,000 fosmids) retrieved by similar means from epipelagic DCM metagenomics libraries (24).

### Ubiquitous, highly abundant, and widespread viruses.

**FIG 3:**
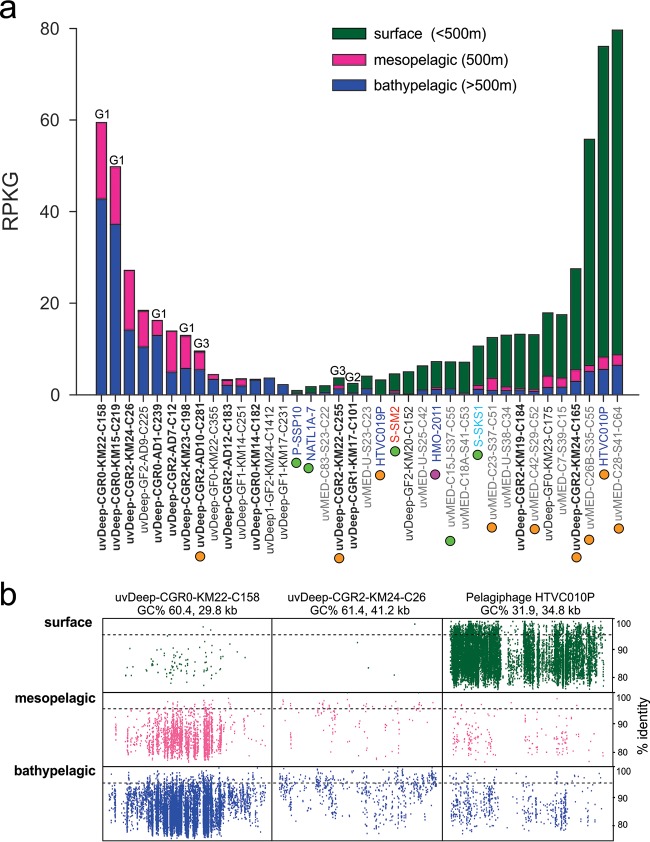
(a) Recruitment of uvDeep genomes and other references from the Pacific Ocean virome (POV). The POV data set was divided in subsets of surface, mesopelagic, and bathypelagic data (<500 m, 500 m, and >500 m deep, respectively). The recruitment is measured in RPKG (reads per kilobase of genome per gigabase of the data set). The known (or predicted) hosts of the viruses are indicated by colored circles (green, cyanophages; orange, SAR11 phages; pink, SAR116 phages). Phage morphology is indicated by the label color (dark blue, podoviruses; red, myoviruses; light blue, siphoviruses). Previously described genomic fragments from the uvMED collection are shown in gray; sequences from this work are shown in black (boldface, complete genomes). A label (G1 to G3) is indicated on the top of the bars of genomes belonging to one of the three groups identified in the complete uvDeep genomes. (b) Recruitment plot of two uvDeep genomes in comparison to pelagiphage HTVC010P, highlighting the differences in abundance levels across a depth profile.

To evaluate the distribution of uvDeep viruses in the water column, we computed the number of reads recruited by each viral contig from the Pacific Ocean virome at different depths: surface, mesopelagic, and bathypelagic (Fig. 3a). Only uvDeep viral genomic fragments recruiting more than 2 RPKG (reads per kilobase of genome per gigabase of metagenome) from three different depths are shown in Fig. 3a. A large number of uvDeep viruses appear to be found exclusively in deep waters, recruiting reads only from meso- and bathypelagic viromes. Moreover, all genomes found exclusively in meso- and bathypelagic waters are uvDeep viruses, further emphasizing their truly bathypelagic nature. The idea of the depth-specific nature of these genomes was also reinforced by analyses of cellular metagenomes from the surface and the deep Mediterranean (see Fig. S4 in the supplemental material). “*Candidatus* Pelagibacter” phage HTVC010P and *Puniceispirillum* phage HMO-2011 are considered the most abundant cultured marine viruses known to date (29, 30). Remarkably, some of the uvDeep viruses recruited as many reads from meso- and bathypelagic viromes as these viruses do in the photic zone. For example, uvDeep genomes uvDeep-CGR0-KM22-C158 and uvDeep-CGR0-KM15-C219 appear to be almost as abundant in deep waters as pelagiphage HTVC010P is at the surface (10, 29). Moreover, the number of reads recruited by most of uvDeep viruses was greater than or similar to the number that HMO-2011 recruited from the surface.

The viral genome uvDeepFos-CGR0-KM22-C158 recruited the greatest number of reads from deeper waters. The recruitment plot in Fig. 3b shows that these genomes recruit along their entire lengths with nucleotide identities in the 80% to 95% range, suggesting that highly related viral genomes are also found in the POV data set. Overall, these results indicate that uvDeep Mediterranean viruses are widely distributed, being present also in the Pacific Ocean. The lack of viromes from deep waters limited the assessment of the distribution of these viruses on a worldwide scale. However, we also searched for the presence and abundance of uvDeep viral sequences in the available metaviromic datasets from the Tara Oceans expedition (31, 32). Again, the uvDeep viruses do not appear to have been abundant in the Tara’s photic zone and DCM viromes, reinforcing their specificity with respect to deep waters (see Fig. S5 in the supplemental material). On the other hand, many known surface-related viruses (e.g., pelagiphage HTVC010P and *Synechococcus* phage S-SKS1) were detectable throughout the water column. This was not unexpected, as the sinking particles provide a continuous flux of surface microbes into the deep. Moreover, these genomes also recruit far more reads from surface than from deep waters, indicating their prevalence in the photic zone.

Host prediction for these highly recruiting deep-sea viruses was successful for two genomes of group 3 (see below for details). Both uvDeep-CGR2-AD10-C281 and uvDeep-CGR2-KM22-C255 have been tentatively predicted to infect SAR11 representatives as shown by sequence similarity to putative uvMED pelagiphages. Phage uvDeep-CGR2-AD10-C281 recruits significantly from meso- and bathypelagic depths, while uvDeep-CGR2-KM22-C255 recruits equally from the two depths (see Fig. S5 in the supplemental material). Although host prediction was unsuccessful for other highly recruiting genomes, their high abundance in viromes obtained from locations as distant as the Pacific Ocean suggests that they probably infect abundant and widespread bathypelagic microbes, having an important role in the bathypelagic realm.

### Host identification: SAR11 bathyphages.

Since most of the host assignment strategies require viral and host genomic sequences (24, 33), the link between host and viruses in deep waters was expected to be even more challenging to find than for uncultured viruses from the photic zone. However, four single-cell amplified genomes (SAGs) belonging to the SAR11 cluster (*Pelagibacteriales* order, *Alphaproteobacteria*) were recently recovered from bathypelagic waters (34). They belong to the SAR11 subclade Ic, known to be found in deep waters. Using the previously described *att*-*int* relationship (10), we were able to identify one complete phage genome (uvDeep-CGR0-AD1-C123) predicted to infect the deep SAR11 SAG AAA218-E13 (34). We identified a 55-bp-long identical hit in a tRNA-containing contig from the SAR11 SAG (*attB* site) and in a region of the phage genome (*attP*) close to the integrase gene (see Fig. S6 in the supplemental material). This highly significant match suggests that this *attB* gene is the insertion site of this phage within the deep SAR11 genome. Although one of the SAR11 SAGs described was found to include a clustered regularly interspaced short palindromic repeat (CRISPR) locus, no hits were found between the spacers and the viral genomes described here. The sequence of this bathypelagic virus differs considerably from those of all previously described pelagiphages (10, 29). However, results of recruitment analyses performed with available viromes and metagenomes suggest that it is not an abundant phage (see Fig. S4).

Additionally, four uvDeep genomes (uvDeep-CGR2-AD10-C281, uvDeep-CGR2-KM22-C255, uvDeep-CGR2-KM24-C165, and uvDeep-CGR2-AD8-C175) were found to be related to genomes originating from the uvMED data set (24) and predicted to infect “*Ca.* Pelagibacter” representatives (see Fig. S2 in the supplemental material).

### Lysogenic viruses in the deep sea.

The eleven contigs that were predicted to represent proviruses could be assigned to diverse microbes, specifically, *Psychrobacter* (*Gammaproteobacteria*), *Flavobacteriia* (*Bacteroidetes*), *Planctomyces* (*Planctomycetes*), and “*Ca.* Pelagibacter” (*Alphaproteobacteria*) (see Table S2 in the supplemental material). For some of these provirus-containing contigs, it was also apparent that the proviral loci recruited from viromes whereas the cellular genomic regions recruited from metagenomes (see Fig. S7). This suggests that these proviruses have very similar virion relatives in the environment and may have an important role in the dynamics of bathypelagic microbial communities.

## DISCUSSION

The abundance of viral genes in metagenomic fosmid libraries from the photic zone was described very early in the short history of metagenomics (4, 35). This finding was later used to retrieve complete viral genomes from size-fractionated planktonic cells from the photic zone that contain naturally replicating viruses (10, 24). It was apparent from the sequences of these fosmids that the cloned DNA derived from cells undergoing a viral lytic cycle. In particular, caudovirales replicate by forming long concatemers in which the complete phage genome is repeated multiple times, thus providing an ideal material for assembly and genome finishing. A number of overlapping contigs (assembled from different sequencing batches) were found for these viruses in particular, reinforcing the idea that these genomes originated from concatemers and were actively infecting cells at the time of sample collection. In the present work, we used a similar approach to retrieve viral genomes from bathypelagic waters. However, we observed lower yields of total viral genomic fragments and complete genomes than were observed in DCM fosmid metagenomic libraries. While we recovered several highly abundant and globally distributed viral genomes, this lower yield in itself suggests differences between the surface and deep waters in viral strategies. Pelagic microbes in the dark ocean encounter much more stressful conditions than their epipelagic counterparts. The absence of light precludes photosynthesis or rhodopsin-based phototrophy, and, although chemosynthesis seems to occur (36), it is likely that a considerable fraction of microbial bathypelagic life is associated with particulate organic matter that provides oases of localized energy sources. One observation derived from a number of studies carried out in surface waters is that viruses are more abundant in the particulate fraction (37, 38). However, our biomass extraction protocol was aimed at collecting pelagic (free-living) cells that might be starving in deep waters. That our protocol was successful was evident from the fact that we recovered several viral genomes that are also quite abundant at locations as distant as the Pacific Ocean but that the frequency of recovery was lower than that seen with the photic zone.

A number of factors may contribute to this incongruity in viral recovery, likely explanations would be a lower number of viruses undergoing the lytic cycle or smaller burst sizes in deeper waters. As we did not find any overlapping contigs in our deep-sea metagenomic libraries, this may also indicate a lower frequency of active viral infections. Moreover, some genomic fragments may also derive from dormant viruses (proviruses) inserted in the host chromosome, from virions attached to the cell surface, or from (single-copy) plasmids as has been observed for thermophilic archaeal viruses (39). Finally, it is also possible that at least some viral DNA from this size fraction in the deep sea might derive in part from particles (washed through from the 5-μl-pore-size filter) representing a population likely submitted to a feast-and-famine regime. Both viruses and their hosts could follow a strategy of resilience, with sporadic bursts of growth that are more effective at these energy-limited oceanic depths.

Taken together, these findings suggest that bathypelagic viruses favor a largely lysogenic strategy that allows viral replication along with that of the highly diluted hosts. Accordingly, we found a number of fosmid clones of mixed viral and cellular origin, and, coupled with the comparatively lower frequency of viral genome recovery, this strongly suggests that, as has been proposed before (40), lysogeny could play a more important role in deep than in surface waters. It has also been suggested recently that lysogeny is favored in situations of very high (“Piggyback-the-Winner”) or very low (“Piggyback-the-Loser”) host densities (41). The deep ocean certainly fits the conditions required for the latter.

The bottlenecks of assessing bathypelagic microbial communities reflect the lack of cultured representatives and genomic information from deep waters. The scarcity of microbial and viral genomes from this environment has constrained efforts at fuller descriptions of novel uncultured viruses. Attempts at host assignment were also highly compromised, as genomic information from both viruses and their hosts was a prerequisite in most strategies devised until now. Despite these caveats, we were able to identify five putative SAR11 viruses, one of which likely infects SAR11 representatives of the Ic subclade. Microbes from the SAR11 clade are highly abundant and widespread throughout the water column. The first cultured representatives of viruses infecting “*Ca.* Pelagibacter” were described a few years ago and were also shown to be the most abundant in viromes worldwide (29). While the novel SAR11 bathyphages described here do not appear to be as abundant, a few of them recruited exclusively from meso- and bathypelagic waters, indicating their adaptation to deep waters. We have also described a few other very abundant and widespread viruses of as-yet-unknown hosts that are likely to infect representatives of either SAR11 or other major deep-sea lineages. These viruses were shown to be as abundant in deep waters as the surface pelagiphages are on the photic-zone viromes, and, considering the scale of the bathypelagic realm, they may be among the most abundant viruses on Earth.

The widespread occurrence of some of these viruses in deep-ocean metagenomes worldwide reflects the relatively homogeneous communities found throughout the world at bathypelagic depths (i.e., everything is everywhere, but the host selects) and reinforces previous work carried out with the host cells (6, 42). The deep Mediterranean water does flow to the global ocean by way of the Gibraltar sill (ca. 400 m deep), and it could guarantee that bathypelagic Mediterranean viruses mix with the deep global ocean via the deep current that links all the deep-water masses (43). The high abundance and wide distribution of these viruses suggest that they may have a major role in carbon shunt, directly influencing global nutrient cycling. Direct sequencing of metagenomic fosmids and single-cell genomics analyses have opened an important route of opportunities for the recovery of complete genomes, providing glimpses at microbes and their viruses that have been inaccessible before and allowing further insights into the ecology and evolution of this unknown majority.

## MATERIALS AND METHODS

### Sample origin.

The viral genomic sequences analyzed in this work were retrieved from two fosmid libraries constructed using DNA purified from the 0.2-to-5-µm-cell-size planktonic fraction from two deep-sea sites in the Mediterranean. The first site is located at a depth of 3,000 m in the Ionian Sea (KM3) (36°29′98″N, 15°39′97″E) and the second at a depth of 1,000 m in the Adriatic Sea (AD1000) (41°36′N, 17°22′E) (6, 12, 14, 44). The metagenomic libraries were constructed using a CopyControl fosmid library production kit (Epicentre) as described in the manufacturer’s instructions and yielded 20,757 and 38,704 clones, respectively. Clones were stored at −80°C in 96-well culture plates. Some results from fosmid end sequencing and complete sequencing of selected clones from these libraries have been described before (6, 12, 14, 44).

### Fosmid sequencing, assembly, and annotation.

For this work, a total of 6,432 fosmid clones (3,168 from AD1000 and 3,264 from KM3) were chosen. DNA was extracted from pools of 48 clones using a QIAprep Spin Miniprep kit (Qiagen, Valencia, CA, USA). DNAs from ~250 fosmids were subsequently pooled to generate a total of 24 batches. These 24 pools were tagged and sequenced (Beckman Coulter Genomics, Fort Collins, CO, USA) using paired-end Illumina HiSeq 2000 (2 by 100 bp) in a single lane (~68 Gb data with ~450 million reads, ~180× coverage for each fosmid, assuming each fosmid length to be ~50 kb). After demultiplexing, sequences from each of the 24 pools were independently assembled using Velvet (45) (version 1.2.03; *k* = 71). All genes were predicted using prodigal (46) and annotated using BLAST against the NR database (E value, <1e−5; >70% hit and query coverage), Pfam (47) (using trusted score cutoffs), COGs (>70% hit and query coverage; E value, <1e−5) (48), TIGRfams (using trusted score cutoffs) (49), and phage orthologous groups (POGs) (>70% hit and query coverage; E value, <1e−5) (50). All viral proteins were also annotated using the HHPred server (51).

### Identification of viral contigs.

Several criteria were used to identify viral genomic fragments. In addition to the preclassification based on the contig annotation, the presence of phage orthologous groups (POGs) was used to identify putative viral contigs. All contigs were manually inspected to confirm their viral origin. Complete viral genomes were identified by the presence of end redundancy in a single contig. These methods have been previously described (10).

### Terminase tree phylogeny.

To collect reference sequences to create the tree of terminase domains shown in Fig. 1, the NCBI NR database was searched for phage large-subunit-terminase-domain-containing proteins (terminase_1, terminase_3, terminase_6, and terminase_GpA) using Pfam HMMs and the HMMER3 package (52). Only those proteins in which the coverage of the domain was >95% were retained, and domain sequences were extracted from the proteins. These were clustered using usearch (53) at a 50% identity level to remove redundancy. However, sequences closely related to the uvDeep terminase domains were retained. Additionally, a total of 152 nonredundant domain sequences (referred to here as the uvMED sequences) were added from a previous publication, while 52 sequences are from the present data set (referred to here as uvDeep). We also included previously described terminases from uncultured viruses from the MedDCM (referred to as uvMED) and from viruses infecting important marine microbes such as *Synechococcus*, *Prochlorococcus*, and “*Ca.* Pelagibacter.” The final set contained 1,119 large terminase domain sequences. These were aligned using an iterative refinement strategy implemented in MAFFT (54). The maximum likelihood phylogenetic tree was computed using FastTree2 (55) with a JTT + CAT model and an estimation of the gamma parameter. Bootstrapping was performed using the Seqboot program in the PHYLIP package (56).

### Genomic comparisons and classification.

The heat map shown in Fig. S1 in the supplemental material was created by comparing the predicted viral proteins from all viral genomes to each other using BLASTP (57) and the BLOSUM45 matrix. A hit was considered conserved if the protein sequence was >30 amino acids (aa) in length, had an E value of 0.01, and showed >30% identity. Only one hit per protein was counted, providing a percentage of conserved hits between two genomes (normalized by self-comparisons). Genomes with >20% conserved hits were considered part of the same group. The complete viral genomes were also compared to reference genomes as described previously (10), and a neighbor-joining tree reflecting the relationship between reference genomes and those obtained here was constructed. Additional comparisons among related viral genomes, selected genomic fragments, and reference genomes were performed using tBLASTx and BLASTN (57).

### Comparative fragment recruitment.

To estimate the abundance and distribution of these novel viruses, we performed fragment recruitment using different marine metagenomes (5, 7) and viromes (10, 20–22, 58). The Pacific Ocean virome (POV) data set was concatenated according to the different depths (for surface data, 16 samples from depths of 5 to 105 m; for mesopelagic data, 4 samples from a depth of 500 m; for bathypelagic data, 12 samples from depths of 1,000 to 4,300 m). Metagenomic reads were compared to viral genomes using BLASTN, and only hits with >95% identity, an alignment length of >50 bp, and an E value of <1e−5 were considered for computing the number of reads recruited per kilobase of genome per gigabase of metagenome (RPKG).

### Host identification.

We compared all viral genomes harboring an integrase against available microbial genomes from the marine environment, especially those from deep samplings, using BLASTN. Only hits with lengths greater than 30 bp and with 100% nucleotide identity were considered. A significant host-virus relationship was confirmed only if the hit in the host genome was coincident with a tRNA and the region in the viral genomes was close to the integrase gene.

### Accession numbers.

All the viral genomic sequences described in this work were deposited in GenBank (accession numbers KT997784-KT997882). All Mediterranean metagenomes that were used for recruitment are available at NCBI under BioProject identifiers PRJNA257723 and PRJNA305355.

## SUPPLEMENTAL MATERIAL

Figure S1  Comparison between uvDeep and uvMED contigs. A few comparisons between the phages recovered from both the photic and the deep datasets are shown (tBLASTx). Phages from the uvMED library are indicated with a blue diamond and those from the uvDeep library with a pink diamond sign. Putative pelagiphages are indicated with orange circles. Download Figure S1, PDF file, 0.04 MB

Figure S2  Heat map of an all-versus-all protein comparison of all complete uvDeep genomes. A color scale representing percent conservation is shown at top right. The three groups with proteins conserved at rates of >20% (group 1, group 2, and group 3) are highlighted in white squares within the matrix and labeled. Download Figure S2, PDF file, 0.2 MB

Figure S3  Genomic comparison of novel, complete phage genomes (CGRs) with known tailed phages. An all-versus-all comparison of all the 28 complete genomes identified in this work with all known marine phage genomes and several reference tailed phage genomes is shown. The genomes have been clustered using a sequence similarity metric (see Materials and Methods). The ICTV (International Committee on Taxonomy of Viruses) nomenclature for several phages is also shown for reference. Complete genomes from this study are represented by pink squares and previously described uncultured marine phage genomes by blue squares. Download Figure S3, PDF file, 0.1 MB

Figure S4  (a) Comparative recruitment of uvDeep genomes and other references from four Mediterranean metagenomes (shown as bars [two datasets from the deep chlorophyll maximum and two from the deep]). The viral genomes are arranged exactly as in Fig. 3a. (b) Comparative recruitment of selected genomic fragments of the uvDeep data set against four Mediterranean metagenomes. The genomic fragments containing proviruses are labeled in brown. Complete genomes from uvDeep are shown in boldface and black, uvDeep genomic fragments (GFs) in lightface and black, uvMED genomes in gray, podoviruses in blue, myoviruses in red, and siphoviruses in light blue. Download Figure S4, PDF file, 1.3 MB

Figure S5  (a) Comparative fragment recruitment of top recruiting uvDeep phages (including deep pelagiphages) versus surface pelagiphage HTVC010P versus the Tara virome mesopelagic data set using samples from a depth of 800 m. (b) Comparative recruitment of uvDeep genomes and other references from Tara viromes. The phage genomes are arranged exactly as in Fig. 3a. Download Figure S5, PDF file, 0.3 MB

Figure S6  Deep pelagiphage uvDeep-CGR0-AD1-C123 genome and the probable insertion point in the genome of SAG SAR11 AAA288-E13. Download Figure S6, PDF file, 0.1 MB

Figure S7  Fragment recruitment plots of contigs representing proviruses of a *Bacteroidetes*, alphaproteobacteria, deltaproteobacteria, and a gammaproteobacteria from selected metaviromic and metagenomic datasets (names are shown at bottom right). Percent identity (nucleotides) data are shown on the *y* axis, and the reads are shown in blue (metagenomes) and red (viromes). Download Figure S7, PDF file, 0.6 MB

Table S1  Complete list of all phage contigs described in this study. Table S1, PDF file, 0.05 MB

Table S2  Complete list of all provirus contigs described in this study. Table S2, PDF file, 0.03 MB
