# Supplementary material for: Genomes of Abundant and Widespread Viruses from the Deep Ocean
Source: mBio. 2016 Jul 26;7(4):e00805-16. doi: 10.1128/mBio.00805-16 (PMC4981710; doi:10.1128/mBio.00805-16)
Supplement: Figure S1 — Comparison between uvDeep and uvMED contigs. A few comparisons between the phages recovered from both the photic and the deep datasets are shown (tBLASTx). Phages from the uvMED library are indicated with a blue diamond and those from the uvDeep library with a pink diamond sign. Putative pelagiphages are indicated with orange circles. Download [file mbo004162901sf1.pdf]

Viruses from the Deep
Fig S1

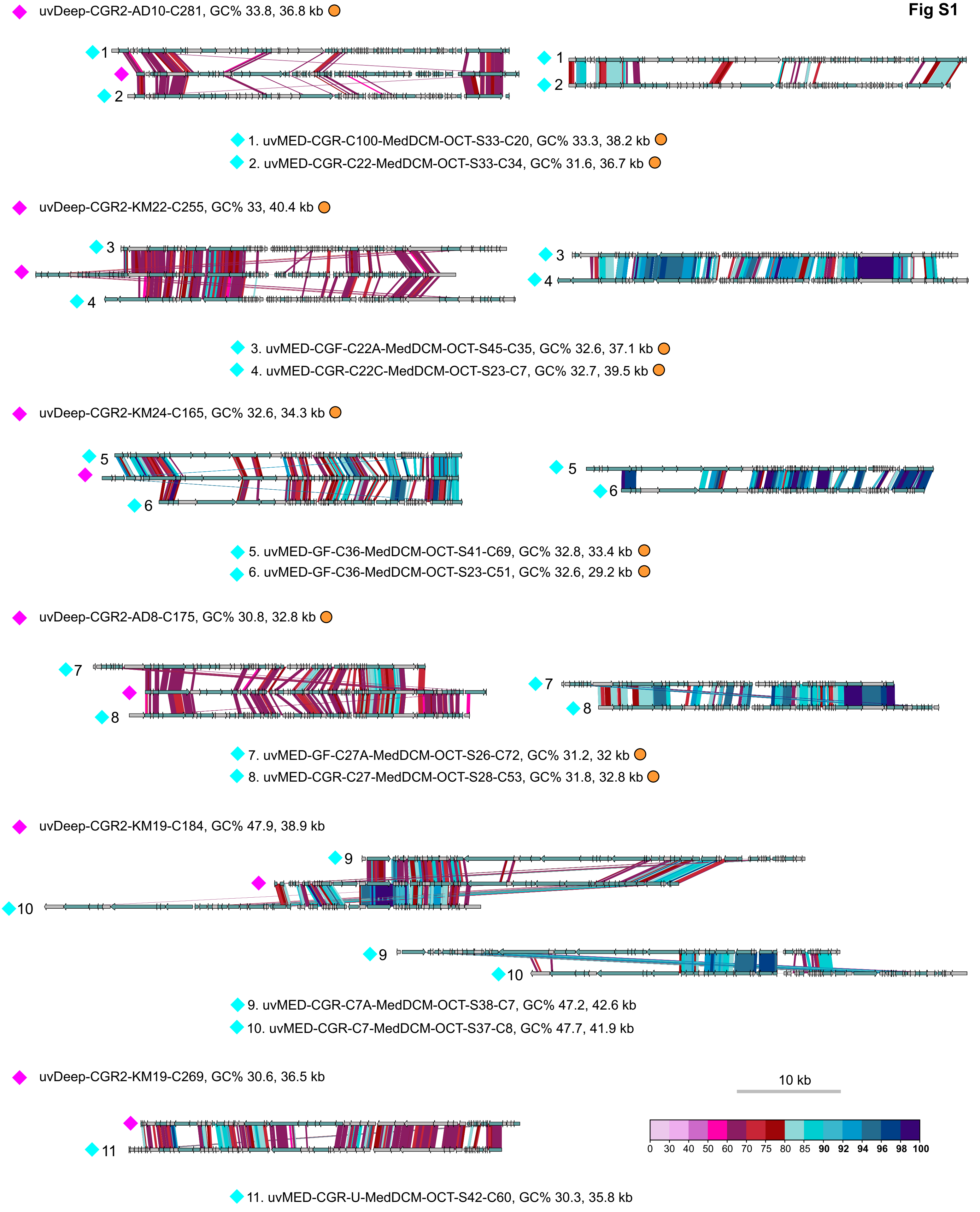

FigS1. Comparison between uvDeep and uvMed contigs. A few comparisons between the phages recovered from both the photic and the deep datasets are shown (tblastx). Phages from the uvMED library are indicated with a blue diamond and those from the uvDeep library with a pink diamond sign. Putative pelagiphages are indicated with orange circles.
