# Supplementary material for: Genomes of Abundant and Widespread Viruses from the Deep Ocean
Source: mBio. 2016 Jul 26;7(4):e00805-16. doi: 10.1128/mBio.00805-16 (PMC4981710; doi:10.1128/mBio.00805-16)
Supplement: Figure S2 — Heat map of an all-versus-all protein comparison of all complete uvDeep genomes. A color scale representing percent conservation is shown at top right. The three groups with proteins conserved at rates of >20% (group 1, group 2, and group 3) are highlighted in white squares within the matrix and labeled. Download [file mbo004162901sf2.pdf]

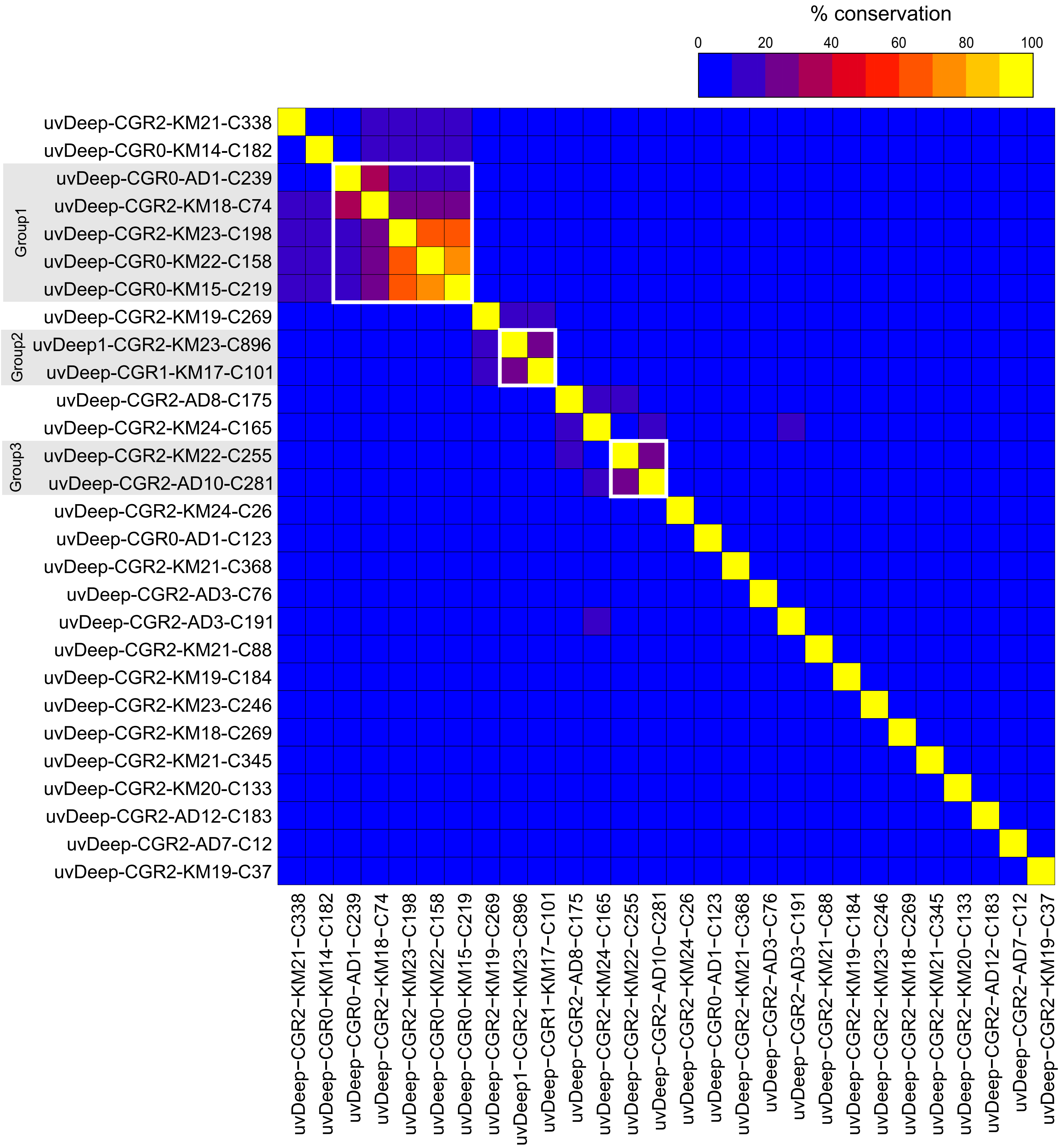

FigS2. Heatmap of an all-vs-all protein comparison of all complete uvDeep genomes. A color scale representing percentage conservation is shown at top right. The three groups, with >20% conserved proteins (Group1,Group2 and Group3) are highlighted in white squares within the matrix and labelled.
