# Supplementary material for: Genomes of Abundant and Widespread Viruses from the Deep Ocean
Source: mBio. 2016 Jul 26;7(4):e00805-16. doi: 10.1128/mBio.00805-16 (PMC4981710; doi:10.1128/mBio.00805-16)
Supplement: Figure S3 — Genomic comparison of novel, complete phage genomes (CGRs) with known tailed phages. An all-versus-all comparison of all the 28 complete genomes identified in this work with all known marine phage genomes and several reference tailed phage genomes is shown. The genomes have been clustered using a sequence similarity metric (see Materials and Methods). The ICTV (International Committee on Taxonomy of Viruses) nomenclature for several phages is also shown for reference. Complete genomes from this study are represented by pink squares and previously described uncultured marine phage genomes by blue squares. Download [file mbo004162901sf3.pdf]

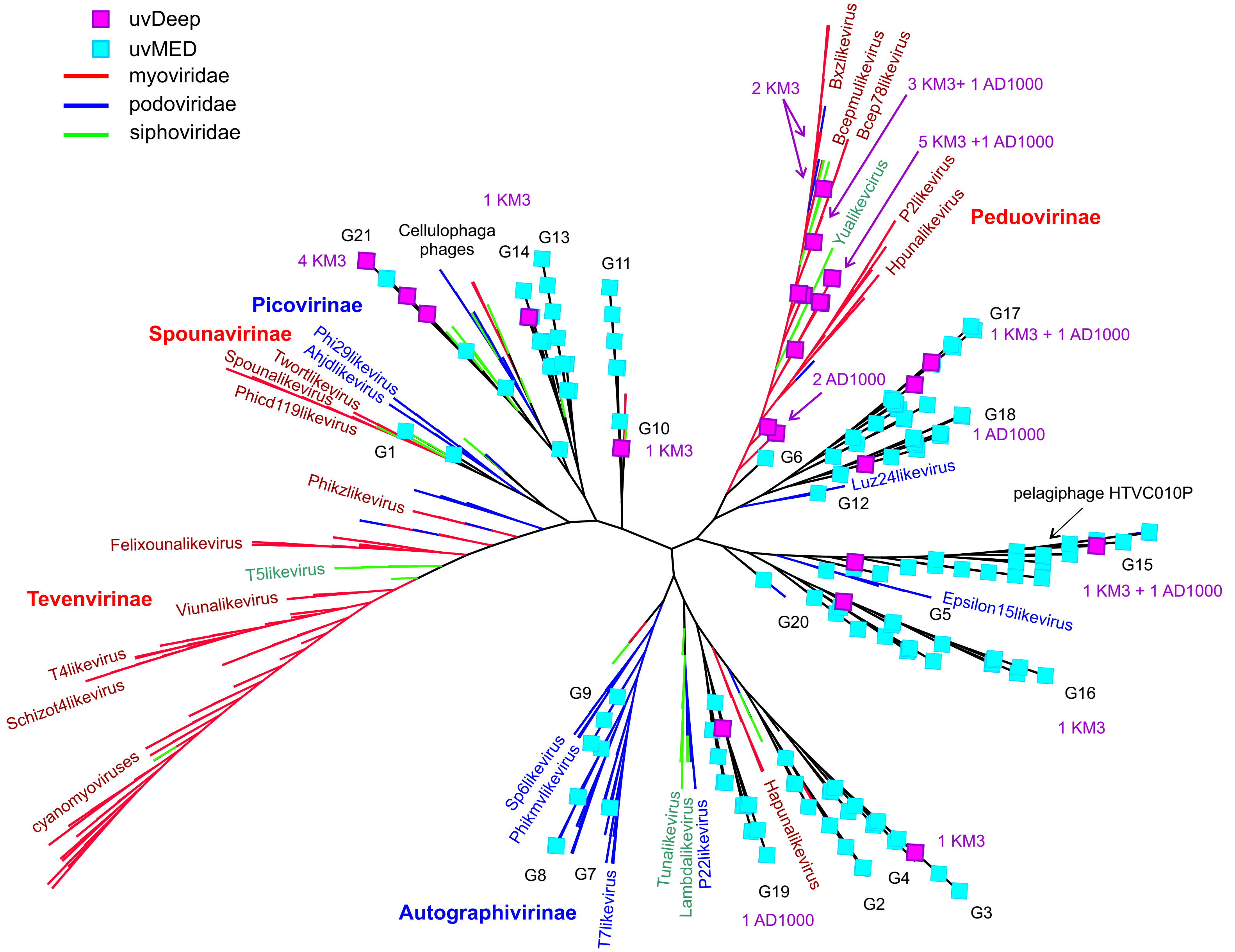

FigS3. Genomic comparison of novel, complete phage genomes (CGRs) with known tailed phages. An all-vs-all comparison of all the 28 complete genomes identified in this work with all known marine phage genomes and several reference tailed phage genomes is shown. The genomes have been clustered using a sequence similarity metric (see methods). The ICTV (International Committee on Taxonomy of Viruses) nomenclature of several phages is also shown for reference. Complete genomes from this study are represented by pink squares and previously described uncultured marine phage genomes in blue squares.
