# Supplementary material for: Genomes of Abundant and Widespread Viruses from the Deep Ocean
Source: mBio. 2016 Jul 26;7(4):e00805-16. doi: 10.1128/mBio.00805-16 (PMC4981710; doi:10.1128/mBio.00805-16)
Supplement: Figure S4 — (a) Comparative recruitment of uvDeep genomes and other references from four Mediterranean metagenomes (shown as bars [two datasets from the deep chlorophyll maximum and two from the deep]). The viral genomes are arranged exactly as in Fig. 3a. (b) Comparative recruitment of selected genomic fragments of the uvDeep data set against four Mediterranean metagenomes. The genomic fragments containing proviruses are labeled in brown. Complete genomes from uvDeep are shown in boldface and black, uvDeep genomic fragments (GFs) in lightface and black, uvMED genomes in gray, podoviruses in blue, myoviruses in red, and siphoviruses in light blue. Download [file mbo004162901sf4.pdf]

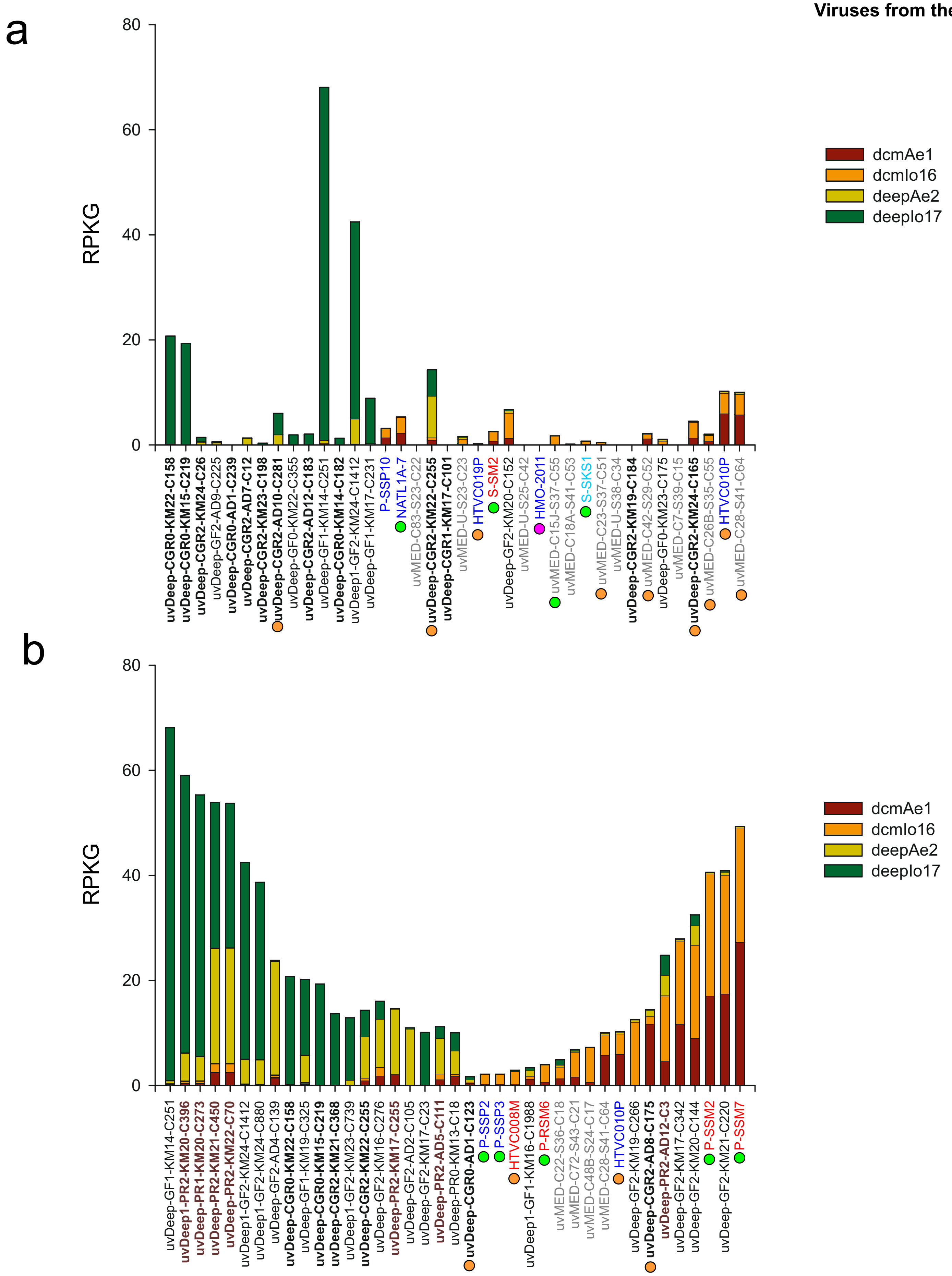

FigS4. a) Comparative recruitment of uvDeep genomes and other references from four Mediterranean metagenomes (shown as bars, two datasets from the deep chlorophyll maximum and two from the deep). The viral genomes are arranged exactly as in Figure 3a. b) Comparative recruitment of selected genomic fragments of the uvDeep dataset against four Mediterranean metagenomes.The genomic fragments containing proviruses are labeled in brown. Complete genomes from uvDeep are bold black (CGRs), uvDeep genomic fragments in black (GFs), uvMED genomes are in grey, podoviruses in blue, myoviruses in red, siphoviruses in light blue.
