# Supplementary material for: Genomes of Abundant and Widespread Viruses from the Deep Ocean
Source: mBio. 2016 Jul 26;7(4):e00805-16. doi: 10.1128/mBio.00805-16 (PMC4981710; doi:10.1128/mBio.00805-16)
Supplement: Figure S5 — (a) Comparative fragment recruitment of top recruiting uvDeep phages (including deep pelagiphages) versus surface pelagiphage HTVC010P versus the Tara virome mesopelagic data set using samples from a depth of 800 m. (b) Comparative recruitment of uvDeep genomes and other references from Tara viromes. The phage genomes are arranged exactly as in Fig. 3a. Download [file mbo004162901sf5.pdf]

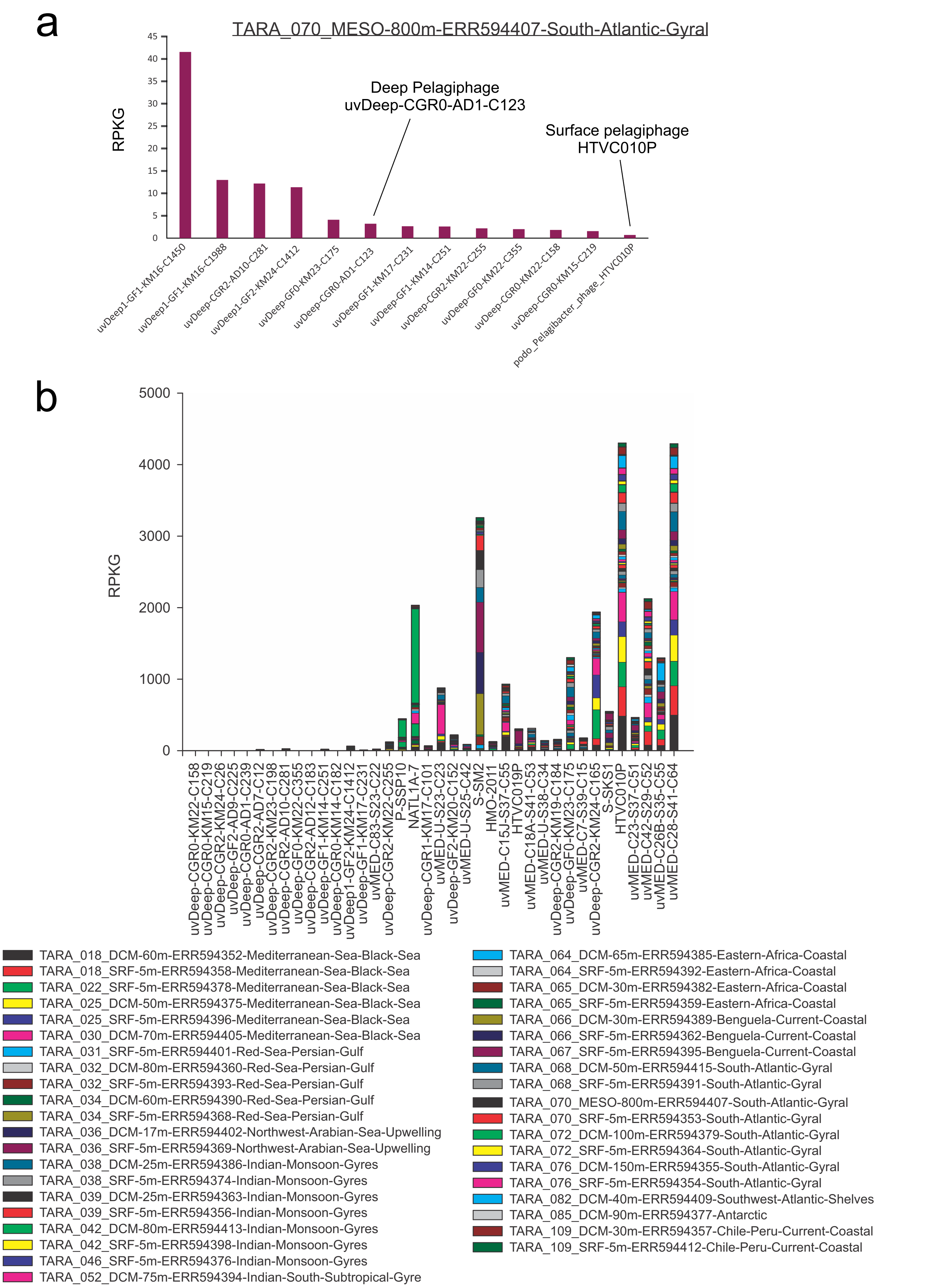

FigS5. a) Comparative fragment recruitment of top recruiting uvDeep phages (including deep pelagiphage) vs surface pelagiphage HTVC010P versus the Tara Virome mesopelagic dataset from a depth of 800m. b) Comparative recruitment of uvDeep genomes and other references from TARA viromes. The phage genomes are arranged exactly as in Figure 3a
