# Supplementary material for: Genomes of Abundant and Widespread Viruses from the Deep Ocean
Source: mBio. 2016 Jul 26;7(4):e00805-16. doi: 10.1128/mBio.00805-16 (PMC4981710; doi:10.1128/mBio.00805-16)
Supplement: Figure S6 — Deep pelagiphage uvDeep-CGR0-AD1-C123 genome and the probable insertion point in the genome of SAG SAR11 AAA288-E13. Download [file mbo004162901sf6.pdf]

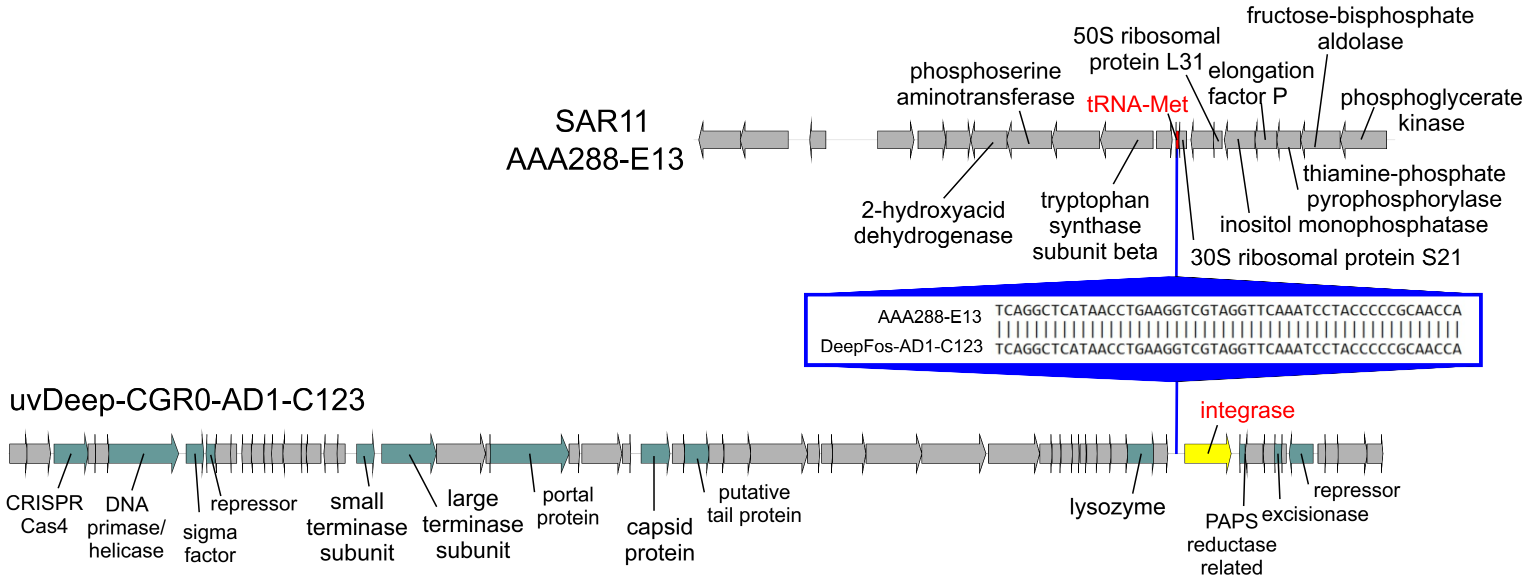

FigS6. Deep pelagiphage uvDeep-CGR0-AD1-C123 genome and the probable insertion point in the genome of the SAG SAR11 AAA288-E13.
