# Supplementary material for: Genomes of Abundant and Widespread Viruses from the Deep Ocean
Source: mBio. 2016 Jul 26;7(4):e00805-16. doi: 10.1128/mBio.00805-16 (PMC4981710; doi:10.1128/mBio.00805-16)
Supplement: Figure S7 — Fragment recruitment plots of contigs representing proviruses of a Bacteroidetes, alphaproteobacteria, deltaproteobacteria, and a gammaproteobacteria from selected metaviromic and metagenomic datasets (names are shown at bottom right). Percent identity (nucleotides) data are shown on the y axis, and the reads are shown in blue (metagenomes) and red (viromes). Download [file mbo004162901sf7.pdf]

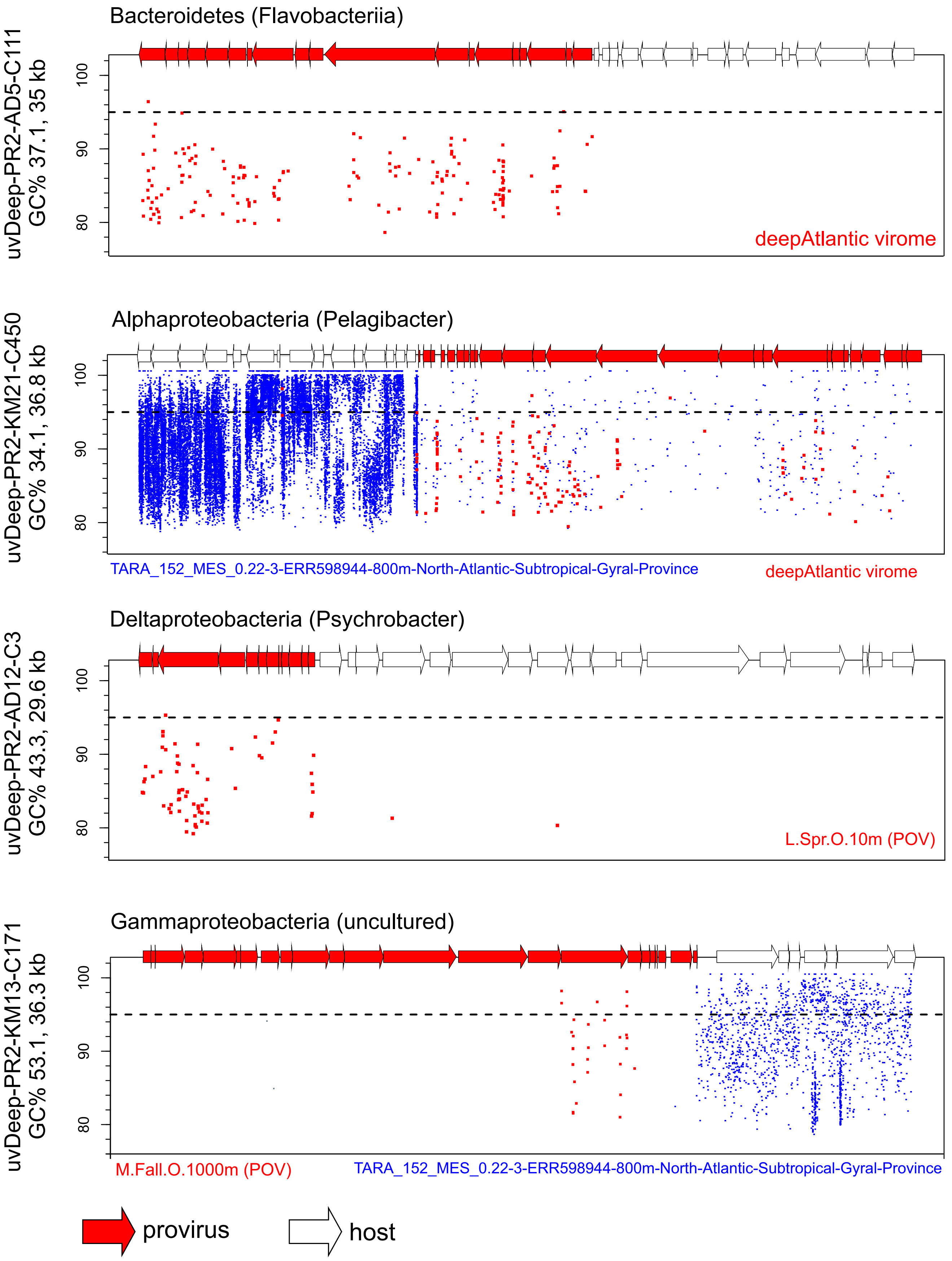

FigS7. Fragment recruitment plots of contigs representing proviruss of a Bacteroidetes, Alphaproteobacteria, Deltaproteobacteria and a Gammaproteobacteria from selected metaviromic and metagenomic datasets (name shown at bottom right). Percentage identity (nucleotides) is shown on the Y-axis and the reads are shown in blue (metagenomes) and red (viromes)
