# Supplementary material for: Genomes of Abundant and Widespread Viruses from the Deep Ocean
Source: mBio. 2016 Jul 26;7(4):e00805-16. doi: 10.1128/mBio.00805-16 (PMC4981710; doi:10.1128/mBio.00805-16)
Supplement: Table S1 — Complete list of all phage contigs described in this study. [file mbo004162901st1.pdf]

Table S1. Complete list of all phage contigs described in this study

	name	length	%GC	contig type	Group	phage type	notes on host
1	uvDeep-CGR2-KM21-C338	34.1	61.94	complete genome		Caudovirales	
2	uvDeep-CGR2-KM24-C26	41.3	61.47	complete genome		Caudovirales	
3	uvDeep-CGR2-AD7-C12	38.0	58.56	complete genome		Caudovirales	
4	uvDeep-CGR2-KM19-C37	36.9	58.54	complete genome		Caudovirales	
5	uvDeep-CGR2-KM20-C133	34.9	60.49	complete genome		Caudovirales	
6	uvDeep-CGR2-KM21-C88	36.0	58.89	complete genome		Caudovirales	
7	uvDeep-CGR0-AD1-C239	34.0	49.39	complete genome	Group1	Caudovirales	
8	uvDeep-CGR0-KM14-C182	39.4	57.07	complete genome		Caudovirales	
9	uvDeep-CGR0-KM15-C219	30.1	60.03	complete genome	Group1	Caudovirales	
10	uvDeep-CGR0-KM22-C158	29.8	60.48	complete genome	Group1	Caudovirales	
11	uvDeep-CGR2-KM18-C74	33.4	45.25	complete genome	Group1	Caudovirales	
12	uvDeep-CGR2-KM23-C198	32.2	53.35	complete genome	Group1	Caudovirales	
13	uvDeep-CGR2-AD12-C183	37.1	53.11	complete genome		Caudovirales	
14	uvDeep-CGR2-AD3-C76	32.2	57.21	complete genome		Caudovirales	
15	uvDeep-CGR2-KM23-C246	37.2	52.53	complete genome		Caudovirales	
16	uvDeep-CGR2-KM18-C269	36.0	37.61	complete genome		Caudovirales	
17	uvDeep-CGR2-AD3-C191	38.1	51.29	complete genome		Caudovirales	
18	uvDeep-CGR2-KM24-C165	34.3	32.57	complete genome		Caudovirales	Alphaproteobacteria
19	uvDeep-CGR2-KM21-C368	36.1	30.95	complete genome		Caudovirales	
20	uvDeep-CGR2-AD10-C281	36.8	33.77	complete genome	Group3	Caudovirales	Alphaproteobacteria
21	uvDeep-CGR2-KM22-C255	40.4	33.01	complete genome	Group3	Caudovirales	Alphaproteobacteria
22	uvDeep-CGR0-AD1-C123	35.6	52.23	complete genome		Caudovirales	Alphaproteobacteria
23	uvDeep-CGR2-AD8-C175	32.8	30.84	complete genome		Caudovirales	Alphaproteobacteria
24	uvDeep1-CGR2-KM23-C896	33.1	30.18	complete genome	Group2	Caudovirales	
25	uvDeep-CGR1-KM17-C101	30.6	34.37	complete genome	Group2	Caudovirales	
26	uvDeep-CGR2-KM19-C269	36.5	30.66	complete genome		Caudovirales	
27	uvDeep-CGR2-KM21-C345	31.6	34.13	complete genome		Caudovirales	
28	uvDeep-CGR2-KM19-C184	38.9	47.88	complete genome		Caudovirales	
29	uvDeep1-GF1-KM16-C1450	17.1	37.15	genomic fragment		Caudovirales	
30	uvDeep1-GF1-KM16-C1988	25.3	32.18	genomic fragment		Caudovirales	
31	uvDeep1-GF2-KM17-C43	34.8	38.45	genomic fragment		Caudovirales	
32	uvDeep1-GF2-KM23-C739	34.0	32.38	genomic fragment		Caudovirales	
33	uvDeep1-GF2-KM24-C1412	38.7	32.39	genomic fragment		Caudovirales	
34	uvDeep1-GF2-KM24-C880	38.0	35.44	genomic fragment		Caudovirales	
35	uvDeep-GF0-AD4-C224	28.0	55.42	genomic fragment		Caudovirales	
36	uvDeep-GF0-KM14-C84	12.7	63.15	genomic fragment		Caudovirales	
37	uvDeep-GF0-KM16-C193	24.6	32.81	genomic fragment		Caudovirales	
38	uvDeep-GF0-KM22-C355	22.9	52.34	genomic fragment		Caudovirales	
39	uvDeep-GF0-KM22-C365	18.5	52.1	genomic fragment		Caudovirales	
40	uvDeep-GF0-KM22-C456	27.9	38.39	genomic fragment		Caudovirales	
41	uvDeep-GF0-KM23-C175	25.2	33.48	genomic fragment		Caudovirales	
42	uvDeep-GF0-KM23-C230	26.2	39.72	genomic fragment		Caudovirales	
43	uvDeep-GF1-AD10-C169	17.6	59.87	genomic fragment		Caudovirales	
44	uvDeep-GF1-AD12-C99	21.0	62.24	genomic fragment		Caudovirales	
45	uvDeep-GF1-AD3-C39	34.0	41.91	genomic fragment		Caudovirales	
46	uvDeep-GF1-KM13-C123	23.5	48.58	genomic fragment		Caudovirales	
47	uvDeep-GF1-KM14-C251	31.6	30.96	genomic fragment		Caudovirales	
48	uvDeep-GF1-KM15-C111	38.1	62.87	genomic fragment		Caudovirales	
49	uvDeep-GF1-KM17-C231	27.6	34.68	genomic fragment		Caudovirales	
50	uvDeep-GF1-KM19-C324	10.8	31.69	genomic fragment		Caudovirales	
51	uvDeep-GF1-KM19-C325	25.4	30.16	genomic fragment		Caudovirales	
52	uvDeep-GF2-AD2-C105	35.9	36.57	genomic fragment		Caudovirales	

continued...

53	uvDeep-GF2-AD2-C266	31.3	52.51	genomic fragment	Caudovirales	
54	uvDeep-GF2-AD4-C139	38.0	33.93	genomic fragment	Caudovirales	
55	uvDeep-GF2-AD5-C185	29.6	49.48	genomic fragment	Caudovirales	
56	uvDeep-GF2-AD7-C162	39.1	32.36	genomic fragment	Caudovirales	
57	uvDeep-GF2-AD8-C17	40.9	41.52	genomic fragment	Caudovirales	
58	uvDeep-GF2-AD9-C225	38.5	62.71	genomic fragment	Caudovirales	
59	uvDeep-GF2-KM16-C11	44.6	57.52	genomic fragment	Caudovirales	
60	uvDeep-GF2-KM16-C276	27.7	36.6	genomic fragment	Caudovirales	
61	uvDeep-GF2-KM17-C119	38.4	36.87	genomic fragment	Caudovirales	
62	uvDeep-GF2-KM17-C191	32.8	38.2	genomic fragment	Caudovirales	
63	uvDeep-GF2-KM17-C202	40.4	53.68	genomic fragment	Caudovirales	
64	uvDeep-GF2-KM17-C23	34.7	39.22	genomic fragment	Caudovirales	
65	uvDeep-GF2-KM17-C280	43.1	46.04	genomic fragment	Caudovirales	
66	uvDeep-GF2-KM17-C342	38.4	35.28	genomic fragment	Caudovirales	
67	uvDeep-GF2-KM18-C120	38.8	35.5	genomic fragment	Caudovirales	
68	uvDeep-GF2-KM19-C237	41.7	53.11	genomic fragment	Caudovirales	
69	uvDeep-GF2-KM19-C266	38.3	34.13	genomic fragment	Caudovirales	
70	uvDeep-GF2-KM20-C144	35.4	33.72	genomic fragment	Caudovirales	
71	uvDeep-GF2-KM20-C152	37.8	35.46	genomic fragment	Caudovirales	
72	uvDeep-GF2-KM21-C220	38.0	34.11	genomic fragment	Caudovirales	
73	uvDeep-GF2-KM21-C24	39.7	45.96	genomic fragment	Caudovirales	
74	uvDeep-GF2-KM24-C13	38.1	57.99	genomic fragment	Caudovirales	
75	uvDeep-GF2-KM24-C45	34.4	58.86	genomic fragment	Caudovirales	
76	uvDeep-GF1-KM20-C130	42.0	45.21	genomic fragment	Iridoviridae	
77	uvDeep-GF0-AD7-C222	18.8	39.24	genomic fragment	Phycodnaviridae	
78	uvDeep-GF0-AD7-C352	20.1	30.95	genomic fragment	Phycodnaviridae	
79	uvDeep-GF1-AD7-C368	12.5	31.38	genomic fragment	Phycodnaviridae	
80	uvDeep-GF1-AD8-C180	19.8	31.78	genomic fragment	Phycodnaviridae	
81	uvDeep-GF1-AD8-C225	15.0	29.91	genomic fragment	Phycodnaviridae	
82	uvDeep-GF1-KM14-C107	20.4	36.92	genomic fragment	Phycodnaviridae	
83	uvDeep-GF1-KM14-C171	18.7	34.08	genomic fragment	Phycodnaviridae	
84	uvDeep-GF1-KM18-C241	10.6	36.09	genomic fragment	Phycodnaviridae	
85	uvDeep-GF1-KM18-C254	12.9	41	genomic fragment	Phycodnaviridae	
86	uvDeep-GF1-KM20-C124	25.5	43.53	genomic fragment	Phycodnaviridae	
87	uvDeep-GF1-KM20-C172	15.7	46.11	genomic fragment	Phycodnaviridae	
88	uvDeep-GF0-KM14-C328	35.7	33.97	genomic fragment	Phycodnaviridae	
89	uvDeep1-PR2-KM20-C396	38.5	31.85	provirus	Caudovirales	Alphaproteobacteria
90	uvDeep-PR0-KM13-C18	30.5	57.84	provirus	Caudovirales	Planctomycetes
91	uvDeep-PR1-AD12-C247	35.2	52.62	provirus	Caudovirales	Alphaproteobacteria
92	uvDeep-PR1-KM20-C273	34.4	32.04	provirus	Caudovirales	Alphaproteobacteria
93	uvDeep-PR2-AD12-C3	29.6	43.32	provirus	Caudovirales	Gammaproteobacteria
94	uvDeep-PR2-AD5-C111	35.1	37.19	provirus	Caudovirales	Bacteroidetes
95	uvDeep-PR2-KM13-C171	36.3	53.14	provirus	Caudovirales	Gammaproteobacteria
96	uvDeep-PR2-KM14-C49	37.3	31.93	provirus	Caudovirales	Alphaproteobacteria
97	uvDeep-PR2-KM21-C450	36.9	34.12	provirus	Caudovirales	Alphaproteobacteria
98	uvDeep-PR2-KM22-C70	36.7	34.14	provirus	Caudovirales	Alphaproteobacteria
