# Supplementary material for: Genomes of Abundant and Widespread Viruses from the Deep Ocean
Source: mBio. 2016 Jul 26;7(4):e00805-16. doi: 10.1128/mBio.00805-16 (PMC4981710; doi:10.1128/mBio.00805-16)
Supplement: Table S2 — Complete list of all provirus contigs described in this study. [file mbo004162901st2.pdf]

**Table S2. Complete list of all provirus contigs described in this study**

| | Contig Identifier | Contig length (Kb) | GC% | provirus length (Kb) | Host Genome |
|---|---|---|---|---|---|
| 1 | uvDeep1-PR2-KM20-C396 | 38.489 | 31.85 | 12.6 | Alphaproteobacteria (*Ca. Pelagibacter* ) |
| 2 | uvDeep-PR0-KM13-C18 | 30.479 | 57.84 | 14.6 | Planctomycetes (Planctomyces) |
| 3 | uvDeep-PR1-AD12-C247 | 35.216 | 52.62 | 17.5 | Alphaproteobacteria (uncultured) |
| 4 | uvDeep-PR1-KM20-C273 | 34.357 | 32.04 | 11.6 | Alphaproteobacteria (*Ca. Pelagibacter* ) |
| 5 | uvDeep-PR2-AD12-C3 | 29.597 | 43.32 | 7 | Gammaproteobacteria ( *Psychrobacter* ) |
| 6 | uvDeep-PR2-AD5-C111 | 35.084 | 37.19 | 20.7 | Bacteroidetes (Flavobacteriia, *Gramella* ) |
| 7 | uvDeep-PR2-KM13-C171 | 36.277 | 53.14 | 27 | Gammaproteobacteria (uncultured) |
| 8 | uvDeep-PR2-KM14-C49 | 37.279 | 31.93 | 28.7 | Alphaproteobacteria (*Ca. Pelagibacter* ) |
| 9 | uvDeep-PR2-KM21-C450 | 36.869 | 34.12 | 23.4 | Alphaproteobacteria (*Ca. Pelagibacter* ) |
| 10 | uvDeep-PR2-KM22-C70 | 36.704 | 34.14 | 23.8 | Alphaproteobacteria (*Ca. Pelagibacter* ) |
